# DLiP-PPI library: An integrated chemical database of small-to-medium-sized molecules targeting protein–protein interactions

**DOI:** 10.3389/fchem.2022.1090643

**Published:** 2023-01-09

**Authors:** Kazuyoshi Ikeda, Yuta Maezawa, Tomoki Yonezawa, Yugo Shimizu, Toshiyuki Tashiro, Satoru Kanai, Nobuyoshi Sugaya, Yoshiaki Masuda, Naoko Inoue, Tatsuya Niimi, Keiichi Masuya, Kenji Mizuguchi, Toshio Furuya, Masanori Osawa

**Affiliations:** ^1^ HPC—and AI-driven Drug Development Platform Division, Center for Computational Science, Yokohama, Kanagawa, Japan; ^2^ Division of Physics for Life Functions, Keio University Faculty of Pharmacy, Tokyo, Japan; ^3^ Udzuki Inc., Tokyo, Japan; ^4^ Lifematics Co., Ltd., Tokyo, Japan; ^5^ PeptiDream Inc., Chiyoda-Ku, Kanagawa, Japan; ^6^ Artificial Intelligence Center for Health and Biomedical Research, National Institutes of Biomedical Innovation, Health and Nutrition, Osaka, Japan; ^7^ Institute for Protein Research, Osaka University, Osaka, Japan

**Keywords:** protein–protein interaction (PPI), database, chemical library, rule-of-4, PMI, drug design, physicochemical property

## Abstract

Protein–protein interactions (PPIs) are recognized as important targets in drug discovery. The characteristics of molecules that inhibit PPIs differ from those of small-molecule compounds. We developed a novel chemical library database system (DLiP) to design PPI inhibitors. A total of 32,647 PPI-related compounds are registered in the DLiP. It contains 15,214 newly synthesized compounds, with molecular weight ranging from 450 to 650, and 17,433 active and inactive compounds registered by extracting and integrating known compound data related to 105 PPI targets from public databases and published literature. Our analysis revealed that the compounds in this database contain unique chemical structures and have physicochemical properties suitable for binding to the protein–protein interface. In addition, advanced functions have been integrated with the web interface, which allows users to search for potential PPI inhibitor compounds based on types of protein–protein interfaces, filter results by drug-likeness indicators important for PPI targeting such as rule-of-4, and display known active and inactive compounds for each PPI target. The DLiP aids the search for new candidate molecules for PPI drug discovery and is available online (https://skb-insilico.com/dlip).

## 1 Introduction

Protein–protein interactions (PPIs) are involved in various biological functions, and over 650,000 PPIs have been identified in the human proteome (Stumpf et al., 2008). PPIs are considered as important targets in drug discovery (Mullard, 2012). However, as the protein–protein (PP) interfaces generally have shallow hydrophobic cavities (Jones and Thornton, 1996; Shin et al., 2017), the hit rate obtained by screening compounds for PPIs using conventional small-molecule compound libraries is not high (Barker et al., 2013). The PP interface includes peptide-binding motifs (Whitby and Boger, 2012), and is therefore structurally complex (Silvian et al., 2013; Milroy et al., 2014; Ran and Gestwicki, 2018). Thus, for targeting PPIs, larger compounds whose chemical space is different from that of conventional small-molecule drugs are required (Reynès et al., 2010; Sperandio et al., 2010; Aeluri et al., 2014; Arkin et al., 2014; Qiu et al., 2020).

Several public databases that contain compound and target information related to PPIs have been released to date, such as TIMBAL (Higueruelo et al., 2013), 2P2Idb (Basse et al., 2016), and iPPI-DB (Labbé et al., 2013). The Protein Data Bank (PDB) (Berman et al., 2000) contains three-dimensional (3D) structural data for PPI complexes, while the ChEMBL database (Gaulton et al., 2012) contains assay data pertaining to PPI inhibition.

However, these open databases lack information on new chemical structures specific to PPIs, because the conventional chemical libraries are composed of small-molecule compounds with molecular weight (MW) < 500, which makes them unsuitable for inhibiting PPI targets. Most molecules that inhibit PPIs are relatively larger than the size recommended by Lipinski’s “rule-of-5 (RO5)” (MW < 500, LogP <5, number of hydrogen-bond donors <5, and number of hydrogen-bond acceptors <10) (Lipinski et al., 1997), and many satisfy the so-called “rule-of-4 (RO4)” (MW > 400, LogP >4, number of rings >4, and number of hydrogen-bond acceptors >4) (Morelli et al., 2011). Therefore, rational design of chemical libraries with new chemical structures is important to find lead compounds that inhibit PPIs. Macrocyclic molecules such as cyclic peptides are considered promising drug candidates against PPIs, while macrocyclic molecules suitable for PPI inhibition suffer from poor cell-membrane permeability (Dougherty et al., 2017). The technologies for generating macrocyclic compound libraries have been advanced (Isidro-Llobet et al., 2011). The chemical synthesis of some macrocycles remains challenging, and those rational designs in the hit-to-lead process are difficult to apply. Therefore, we focused on medium-sized compounds with non-cyclic structures, which have superior synthetic accessibility. Several PPI-oriented libraries have been proposed by academic groups and chemical suppliers (Hamon et al., 2013; Milhas et al., 2016; Bosc et al., 2020). Recently, the Fr-PPIChem library (10,314 compounds) was developed using a machine learning method. However, these chemical spaces are still not wide enough to identify novel PPI modulators. Moreover, molecules complying with the RO4 are often unfavorable as oral drugs owing to their relatively large molecular size, and thus, experimental validation data with reference to PPI targets are lacking for these molecules.

As a part of the Japan Agency for Medical Research and Development (AMED) project, we generated a chemical library (named the DLiP-PPI library) consisting of 15,214 small-to-medium-sized molecules that target PPIs. The library consists of relatively soluble synthetic compounds with MWs ranging between 450 and 650 and was designed to fill the gaps in the chemical space suitable for PPI inhibition. In a previous study, we have demonstrated the usefulness of the DLiP-PPI library by using this library to successfully identify hit compounds with inhibitory activity against Keap1/Nrf2, a PPI target. Moreover, we confirmed the effectiveness of combining the DLiP-PPI library and machine learning in improving the hit rate (Shimizu et al., 2021). However, a relational database (RDB) to store and manipulate the compound data was lacking; thus, the library could not be easily utilized for molecular design.

In this study, we developed a new RDB system and web interface for the DLiP-PPI library. This database is searchable for information on both known PPI modulators and PPI library compounds. It contains a large number of potential PPI modulators with novel chemical structures not found in other data resources, as well as active and inactive compounds linked to a list of known PPI targets that have been originally curated and integrated. In addition, we have implemented some advanced features that are useful for PPI drug discovery such as drug-likeness filters. This database system is useful for designing new compounds for PPI drug discovery research.

## 2 Materials and methods

### 2.1 Preparation of the PPI compound library

The DLiP-PPI library in this database was selected from compounds predicted to bind to 3D structures of various PP interfaces using docking simulations. In addition, we selected additional compounds based on trends in the physicochemical properties of known PPI modulators. To ensure diversity of the PPI targets, the PP interface was classified into three PPI types related to secondary structures (helix, turn, and strand) and other features (motif). First, docking simulations were performed using FRED software (McGann, 2011) on a total of 117 specific PPI-target structures in the PDB for approximately 6 million compounds obtained from a commercial virtual library (K-Library developed by the Kishida Chemical Co. Ltd.). Second, we prioritized compounds based on docking scores and performed clustering and visual inspection to select candidates for subsequent synthesis. Third, to ensure the quality and diversity of the compound library, compounds with a sphere-like structure (*i.e.*, non-flat structure) were also selected by considering the principal moment of inertia (PMI). In addition, new structures were carefully chosen, followed by the selection of novel scaffolds with spiro rings (*i.e.*, new structure) from the virtual compound library. Finally, only compounds that were successfully synthesized were registered in the database (detailed procedures in Supplementary Data Sheet S1).

### 2.2 Preparation of curated protein–protein interaction data

Known PPI-modulating compound and activity data were collected from open PPI databases (TIMBAL, iPPI-DB, and 2P2Idb). Activity data related to known PPI targets were extracted from ChEMBL (version 29), a database of bioactive compounds. In addition, we manually extracted new PPI activity data pertaining to 827 compounds from 14 references published from January 2018 to the present, as these databases did not include these compounds (see references in Supplementary Table S1). Each compound was linked to the PPI-target name (PPI pair) and the activity status (active or inactive). In this database, the PPI-related compounds were classified as active (1) or inactive (0) in a binary manner, and the definition of active or inactive was based on the information derived from the underlying activity information from the original paper. Molecules obtained from TIMBAL and ChEMBL with activity values ≤10 μM were defined as active, and those with higher activity values were defined as inactive. Furthermore, only data in active compounds were extracted from iPPI-DB and 2P2Idb.

## 3 Results and discussion

### 3.1 Contents

**FIGURE 1:**
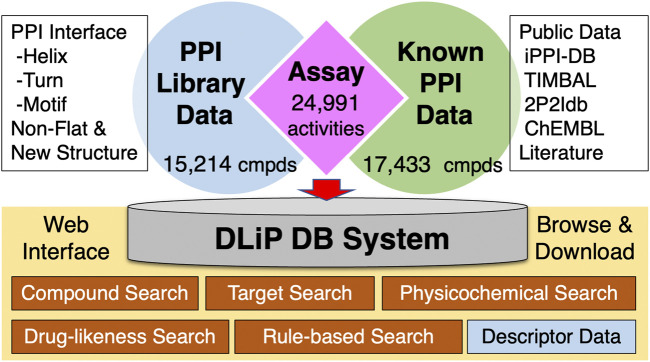
Architecture of the DLiP database system. PPI: protein–protein interaction; cmpds: compounds.

The DLiP database contains a total of 32,647 compounds, comprising 15,214 compounds from the DLiP-PPI library and 17,433 known PPI-related compounds (active and inactive molecules) extracted from public databases and literature (Figure 1). The compound types based on the PP interface being targeted are as follows: helix (α-, 3^10^- and π-helix), 4,908 compounds; turn (β- and γ-turn) and strand (β-strand), 2,566 compounds; and motif, 4,511 compounds. In addition, 2,280 and 949 compounds with non-flat and novel scaffold structures, respectively, were registered. The inhibitory activity data against a PPI target (Nrf2/Keap1) has already been tested for 116 compounds in this library (Shimizu et al., 2021) and then incorporated into the database. The 17,433 known PPI-related compounds were associated with a total of 24,991 activity datapoints (15,129 active and 9,862 inactive datapoints) against 105 PPI targets.

### 3.2 Analysis

**FIGURE 2:**
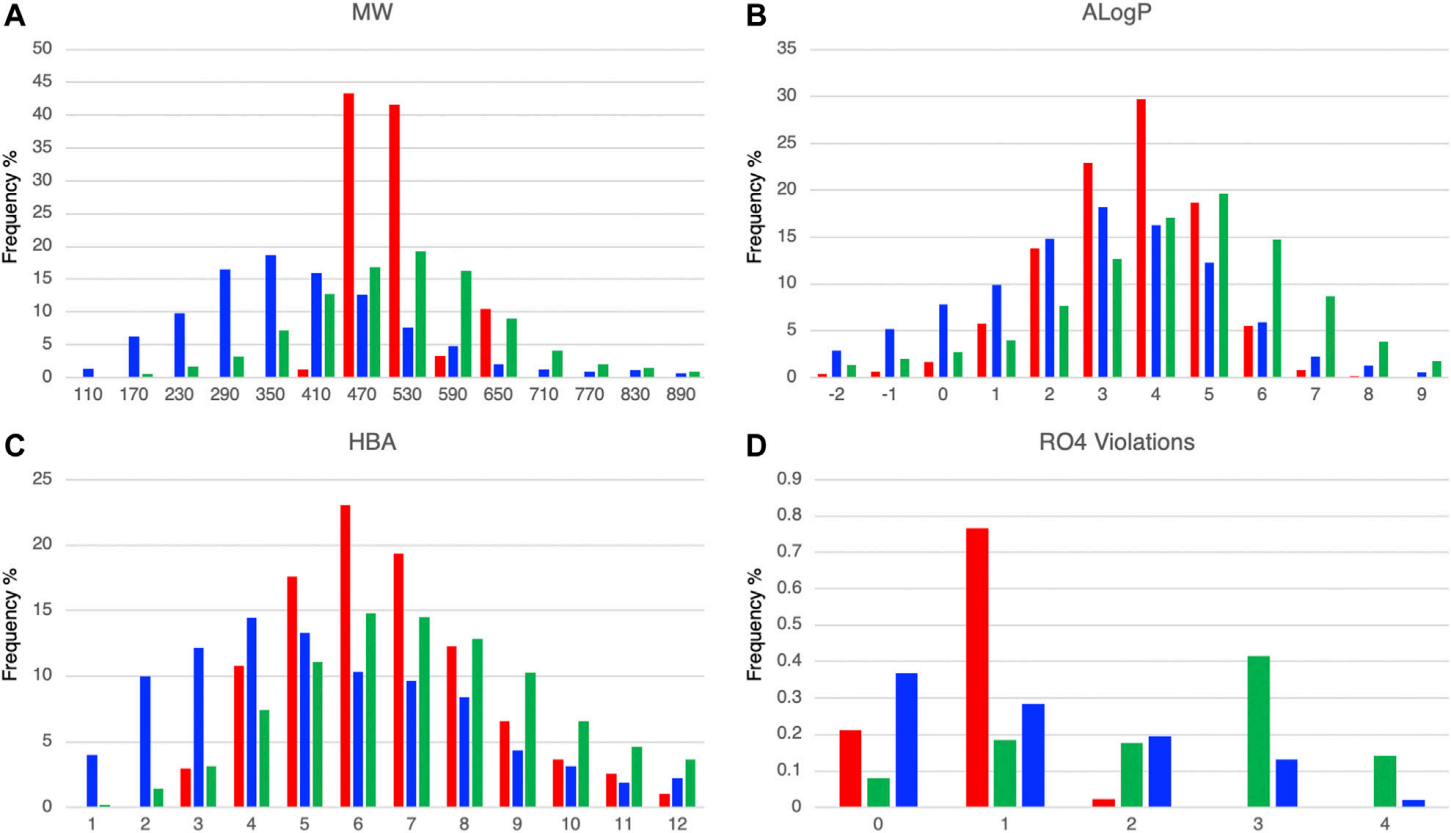
Comparison of histograms of molecular properties (molecular weight **(A)**, ALogP **(B)**, hydrogen-bond acceptors **(C)**, number of violations of rule-of-four **(D)**) of DLiP-PPI library (red), small-molecule approved drugs (blue), and known PPI modulators (green).

We calculated the molecular properties of the compounds in the DLiP-PPI library (15,214 compounds) and known PPI modulators (10,568 compounds) in the database and compared them to approved small-molecule drugs (2,081 compounds). Figures 2A, B show the distribution of representative molecular properties (MW and ALogP) of the compounds in the DLiP-PPI library and known PPI modulators. Majority of the compounds in the DLiP-PPI library (14,448/15,214 = 0.949) have MWs greater than 450 (mean value of 491.28), which is larger than the mean MW value of approved small-molecule drugs (364.67) and closer to the average of that for known PPI-active compounds (524.73). On the other hand, the distribution of ALogP in the DLiP-PPI library (mean value of 3.03) is clearly shifted to a region lower than that corresponding to known PPI modulators (mean value of 3.58), suggesting that these compounds have advantages with respect to oral bioavailability. The DLiP-PPI library consists of synthetic compounds with relatively low ALogP, although the upper MW is limited to approximately 650. The reason for the average MW of this PPI library being lower than that of known PPI modulators is that we left sufficient room in chemical space for structural expansion in the hit-to-lead process. The distribution peaks of hydrogen acceptors, one of the key properties for PPI targeting, are between 6 and 7 for known PPI modulators and the DLiP-PPI library, which are comparable between the two compound groups (Figure 2C and Supplementary Table S2). The number of RO4 violations clearly indicates the suitability for PPI targeting among the datasets (Figure 2D); the distribution of RO4 violations for the DLiP-PPI library is predominantly within one violation. This is comparable to the distribution pertaining to PPI modulators rather than to that of small-molecule drugs, suggesting a potentially advantageous property as a match for PPI targets.

**FIGURE 3:**
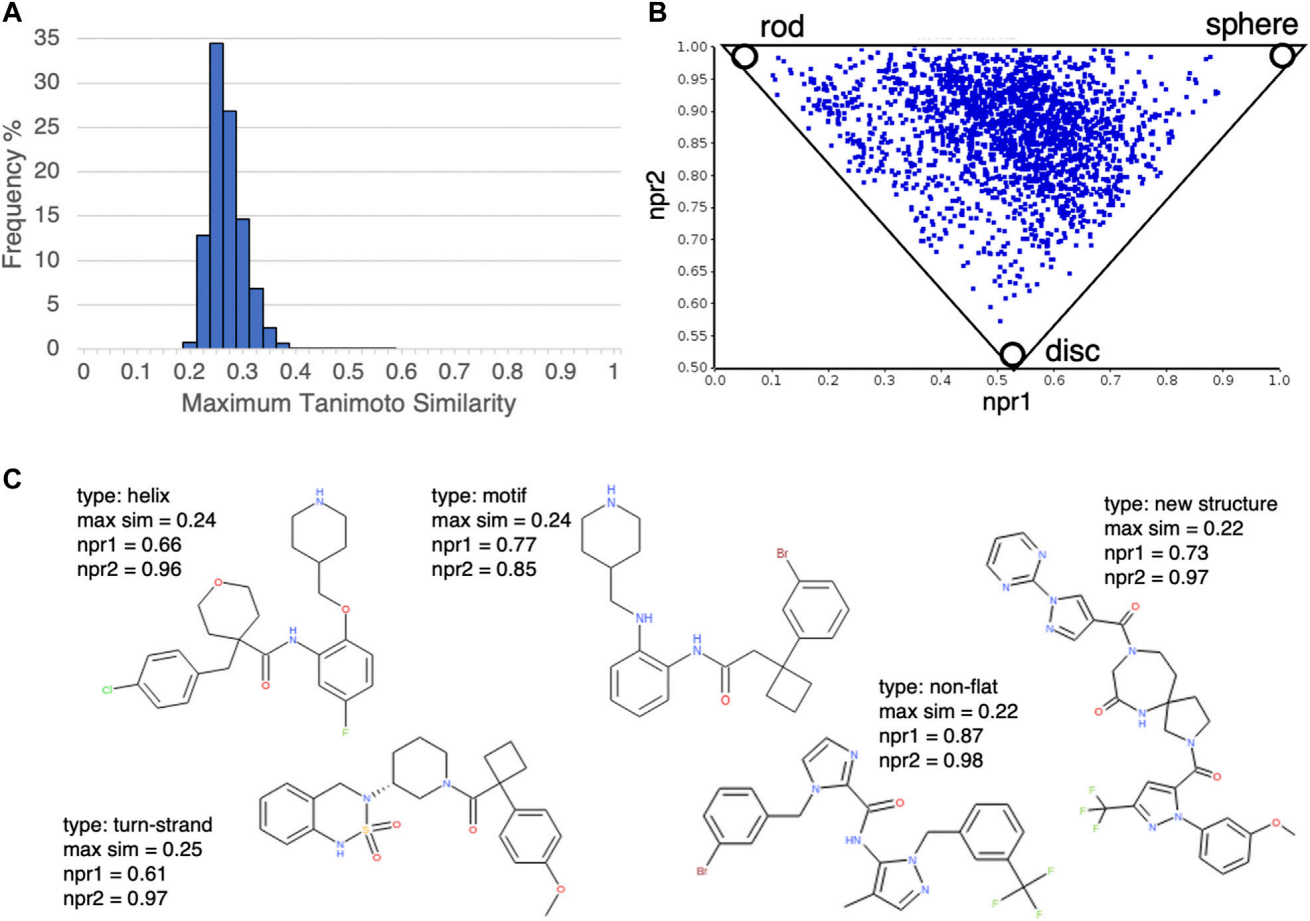
**(A)** Histogram of structural similarities (Tanimoto similarity by extended connectivity fingerprint 6 (ECFP6)) between compounds in the DLiP-PPI library and the closest known PPI modulators in the database. **(B)** Analysis of the steric shape space of non-flat subset of DLiP-PPI library (2,280) by PMI plot. To assess the shape-based distribution of compounds in the PPI library, normalized principal moments of inertia (NPR1 and NPR2) were calculated. **(C)** Examples of compound structures in the DLiP-PPI library. Compounds with npr1+npr2 values greater than 1.5 and maximum similarity to the closet known PPI modulators less than 0.3 were selected. The PP interface type (type), maximum similarity score (max sim), npr1and npr2 are also shown.

Next, we investigated the novelty of the compound structures included in the database. According to our calculation of the Tanimoto similarity between the DLiP-PPI library compounds and known PPI-active molecules using extended connectivity fingerprint 6 (ECFP6) (Rogers and Hahn, 2010), the distribution of maximum similarity scores was mostly less than 0.4 (Figure 3A); this suggests that the novelty of the DLiP-PPI library is high. A triangle plot of normalized PMI ratios (NPR1 and NPR2) gives a visual representation of the molecular shape diversity covered by a collection of molecules (Sauer and Schwartz, 2003). We used the PMI plot to assess the molecular shape-based distribution of compounds in the PPI library. The PMI plot in Figure 3B shows that the compounds in the non-flat subset of the DLiP-PPI library (2,280 compounds) are spread across the region between the sphere-like corner and the disc-rod axis in the PMI space, suggesting high diversity of the steric shapes. The ring structures that occur frequently in each dataset are presented as a list of structures in Supplementary Figure S1 and Supplementary Figure S2. This information may be useful in understanding the typical substructures of PPI modulators or for searching new molecule designs. Notably, the DLiP-PPI library contains unique compounds with the spiro scaffold which is found rarely in the other databases (Figure 3C).

### 3.3 Web interface

**FIGURE 4:**
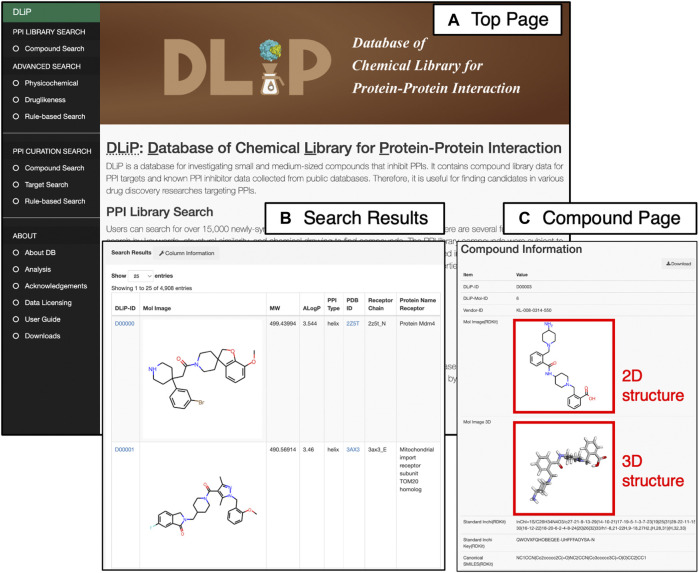
Web interface of the DLiP database system showing **(A)** Top page of the DLiP web interface. Here, users can choose from two main search functions (PPI Library Search and PPI Curation Search) and obtain other information about the DLiP database (ABOUT) from the left menu bar. **(B)** Search result page of PPI Library Search. **(C)** Compound information page. Here, users can see the 2D/3D chemical structures of each compound registered in the DLiP database.

The DLiP database system has a user-friendly web interface that allows users to easily search for PPI-related compound data. It is equipped with two main search functions: the “PPI Library Search” for searching the DLiP-PPI library and the “PPI Curation Search” for searches pertaining to known PPI modulators (Figure 4). In addition, several search options allow users to further filter compounds by physicochemical properties or rule-based drug-like measures. Once users find the compounds of interest, the detailed information is displayed on the “Compound Information Page” that shows chemical structure data (standard InChI, InChIKey, SMILES), typical molecular properties, PPI activity (active or inactive), target information, and descriptors calculated by RDKit 2018.03.4.0 (https://www.rdkit.org/), Mordred 1.1.1 (Moriwaki et al., 2018), and CDK 1.1 (Willighagen et al., 2017). Requests for chemical synthesis for all of the compounds can be placed using the vendor’s compound ID. A total of 1,911 descriptors have been stored in this database.

#### 3.3.1 PPI library search

**FIGURE 5:**
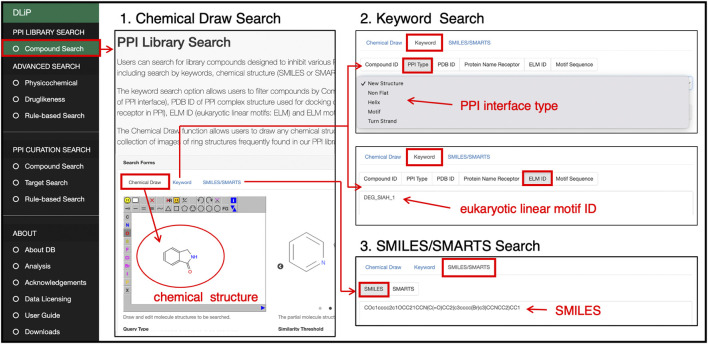
PPI library search page at which users can search for compounds in the PPI library by drawing a chemical structure, selecting or entering a keyword such as PP interface type, motif sequence or ID, or a SMILES/SMARTS string of a chemical structure.

Users can search for compounds included in the DLiP-PPI library by keywords, chemical strings (SMILES and SMARTS), and chemical drawings (Figure 5). In the keyword search, a PDB ID of the PPI-target complex used in docking simulations for PPI modulator prediction, receptor or peptide name, type of secondary structure or motif of the PP interface (PPI type), motif ID (Eukaryote Linear Motif, ELM ID) (Kumar et al., 2019), and motif sequence can be entered to search for compounds. The advanced search functions allow users to filter compounds by physicochemical or drug-likeness properties. The initial positions of the two cursors in the physicochemical search indicate 25% and 75% interquartile deviations for each chemical property in the library. The drug-likeness search allows users to filter compounds from the PPI library by RO5, QED drug-likeness score (Bickerton et al., 2012), and fraction of sp^3^ carbon atoms.

#### 3.3.2 PPI curation search

**FIGURE 6:**
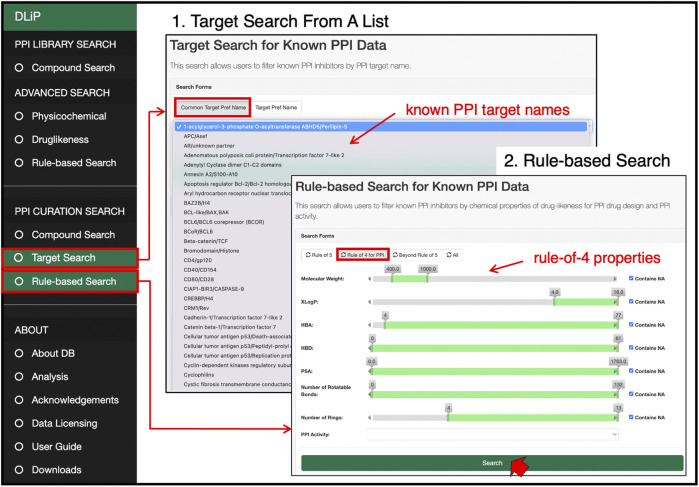
PPI curation search page at which users can select a target of interest from a list of 105 PPI target names, and search for PPI-related compounds by pre-defined drug-likeness rules such as Lipinski’s rule-of-5, rule-of-4, and beyond-rule-of-5.

Users can search for known PPI inhibitors by keywords, chemical strings (SMILES and SMARTS format), and chemical drawings (Figure 6). The target search can be applied by selecting a specific PPI target from the list of PPI-target names. The list of PPI targets in this database was created by curating and integrating targets registered under different target names in several public databases into a common PPI-target name. By using the common PPI-target name, users can easily find compounds related to the same PPI target from different data sources. These compounds are labeled with activity information (active or inactive) for the selected targets. If the users are only interested in compounds favorable for inhibiting PPIs, a drug-likeness search can filter compounds satisfying the RO4 (MW > 400, ALogP >4, number of rings >4, and number of hydrogen-bond acceptors >4).

#### 3.3.3 Search example and case study

Using this interface, users can search for trends among the known PP modulators. Users can easily find a specific target from a PPI list in the PPI curation search page, and then obtain the related compounds and the corresponding physicochemical properties on the compound information page. Alternatively, users can find new chemical structures for a particular PPI target by searching the curated data in this database; such a search yields chemical structures from the search results of the PPI-active compounds of interest. Furthermore, by inputting the substructure into the substructure search function, it is possible to find new compounds from the PPI library with substructures similar to those of the specific PPI target.

#### 3.3.4 Schema and future improvements

The DLiP database system is accessible to anyone at https://skb-insilico.com/dlip. Details of this database schema are shown in Supplementary Figure S3. In the future, we will import additional PPI library compounds as well as supplementary experimental data on PPI inhibition. We will implement functionality in the interface that allows pre-registered users to submit the results of their PPI experiments and all guest users to view the submitted compounds and activity data. Furthermore, one of the goals of this database is to apply it to computational chemistry, and we plan to implement a function to download the descriptor data in a format that can be used as input for building prediction models.

## 4 Conclusion

The DLiP database system was developed to facilitate searching of chemical structures and molecular descriptors related to PPI-oriented chemical libraries. This PPI-oriented library contains unique synthetic compounds with novel chemical structures designed based on the structures of different PP interfaces. Known PPI-related compound data associated with 105 PPI targets were collected from public databases and literature sources and then integrated into this database. Furthermore, by using the web interface, users can easily filter compounds of interest from different data sources according to the RO4, which is an important index for PPI modulators. This database may provide new clues for PPI drug discovery by filling gaps in chemical data between those for small- and medium-sized molecules.

## Data Availability

Publicly available datasets were analyzed in this study. This data can be found here: https://skb-insilico.com/dlip.
