# Supplementary material for: DLiP-PPI library: An integrated chemical database of small-to-medium-sized molecules targeting protein–protein interactions
Source: Front Chem. 2023 Jan 9;10:1090643. doi: 10.3389/fchem.2022.1090643 (PMC9868583; doi:10.3389/fchem.2022.1090643)

Supplementary Material

Content

Supplementary Methods

Supplementary Table 1

Supplementary Table 2

Supplementary Figure 1

Supplementary Figure 2

Supplementary Figure 3

Supplementary Methods. The detailed procedures and concepts underlying the construction of the DLiP protein–protein interaction (PPI) library.

The DLiP-PPI library is a protein–protein interaction (PPI)-oriented library, and is a product developed as part of the Japan Agency for Medical Research and Development (AMED) research project. This library was designed based on three-dimensional (3D) structures of PPI interfaces and physicochemical properties of known PPI inhibitors. We selected the compounds from a virtual compound library for a diverse set of PPI targets using a structure-based drug design (SBDD) approach. Finally, only compounds that were successfully synthesized were registered in the DLiP library.

The procedures were as follows (tools and databases used in this study, Table S1):

1 Selection of target proteins

In the protein complex aspect of the PDB, PPI is conveniently expressed as peptide/protein, where "protein" indicates a receptor protein and "peptide" indicates a peptide sequence binding to the receptor protein. We selected target peptide and protein complex structures from the Protein Data Bank (PDB[1]) (Berman et al., 2000) based on two approaches: i) secondary structure (helix, turn, or strand) of peptides and ii) sequence motif pattern of peptides. First, we collected PDB entries that satisfied the following criteria: amino acid sequence of peptide ≤ 20 residues and of receptor protein ≥ 50 residues. X-ray crystal structures with a resolution > 2.5 Å and structures resolved using nuclear magnetic resonance (NMR) were discarded.

We extracted the secondary structures of the peptides using Define Secondary Structure of Proteins (DSSP) (Kabsch and Sander, 1983) and Structural identification (STRIDE) (Frishman and Argos, 1995). If the sum of frequencies of "H (α-helix),")," "G (3_{10}-helix)," and "I (π-helix)" of a peptide (using DSSP) exceeded 0.2 and the helix region of the peptide contacted a receptor protein, the complex structures of the peptide and receptor protein were treated as helix-focusing target groups. If STRIDE showed that the peptide had turn (β-turn and γ-turn) and strand (β-strand) regions at the interface, we designated peptide/protein complexes as turn-and-strand-focusing target groups from the remaining structures. When any secondary structures were detected in a peptide using DSSP and STRIDE, we searched for sequence motifs located at the protein/peptide interface. Motif patterns registered in the Eukaryotic Linear Motif (ELM) database[2] were used for the search. If any ELM motif in a peptide was in contact with a protein domain of a receptor protein that was registered in ELM as an interacting domain of the motif, we designated the complexes as motif-focusing target groups. Information on protein domains were extracted from Pfam database[3]. To avoid bias in favor of redundant protein domains, the occurrence of each domain was restricted to four over all the target groups.

For each target group, highly similar structures with a sequence identity ≥ 50% between the receptor-proteins were clustered together. We selected 0–2 members from each cluster after assessing the druggability of receptor proteins with respect to pockets located on peptide-binding interfaces. We used the Alpha Site Finder in the Molecular Operating Environment by Chemical Computing Group (MOE[4]) to detect pockets. The druggability scores of all pockets identified on the interfaces were calculated using a machine-learning based method (Sugaya and Ikeda, 2009). We selected peptide/protein complexes with pockets with the highest or the second highest score as representatives

of a cluster. Peptide/protein complexes were discarded when no pockets were identified on the interface or overlapped with the secondary structure/sequence motif of the peptide.

After visual inspection of the complex structures by experts in computer-aided drug design, 117 complexes were finally selected as targets of the docking calculation. Each target group contained 49, 30, and 38 helix-, turn-and-strand-, and motif-focusing complexes, respectively (Tables S2–S4). The most druggable pocket on each peptide/protein interface was used for the docking calculation.

2 Selection of compounds by docking calculation

Compound selection was performed based on a virtual screening study using the docking method. Twice the number of finally expected compounds were selected to account for difficulties in synthesis.

2.1 Preparation of the PPI target

Docking pockets were chosen as follows: 49, 30, and 40 docking pockets for helix, turn-and-strand, and motif mimetics, respectively. The protein structures were downloaded from PDB and preprocessed using the MOE structure preparation tool to clean up resolve any issues; Protonate3D was applied to add protons as appropriate. Then Open Eye Docking Tool Kit[5] was used to create the grid pertaining to the compound-docking pocket for each target.

2.2 Preparation of the compound library

The virtual K-Library was used for docking. We previously worked on this program for several years; as the K-Library was updated two times during that period, we used the latest version each time. K-Library2015-1 and K-Library2015-2 (3.3 and 6.6 million compounds, respectively) were used for helix mimetics, K-Library2016-2 (4.4 million compounds) was used for turn-and-strand mimetics, and K-Library2015-2 was used for motif mimetics. The filtering steps applied to these libraries are listed below.

i) Desalting

ii) Identifying 450 ≤ molecular weight ≤ 900 compounds

iii) Fsp3[6] ≥ 0.356

iv) Repellent structure filtering (Rishton, 1997)

v) PAIN filtering[7]

vi) Exclusion of predicted insoluble compounds[8]

vii) BlockBuster filtering[9]

viii) Deduplication using PPI ChemLib

The molecular weight (MW) was filtered using step ii) because that process targeted PPIs. Considering that PPI inhibitors tend to have spherical and branched shapes (Bosc et al., 2017), the ratio of sp3 carbons was set to that mentioned in step iii) (Zhang et al., 2014; Labbé et al., 2013; López-Vallejo et

al., 2012). The filters in steps iv–vi) and vii) were aimed at excluding problematic compounds and choosing compounds with good properties; step viii) was applied to exclude already marketed libraries. After applying all the filters, 3D structures were generated using Flipper[10], and then the conformers of each structure were generated using the Omega[11] software. Note that compounds previously selected for the lists were removed to avoid duplications between the lists.

2.3 Docking calculation

The FRED[12] software was used for docking the compounds from step **2.2** against the targets from **Subsection 2.1.** Typically, one kind of docking score was used to evaluate compounds; however, we used the consensus score (Charifson et al., 1999) as the ranking power of the scoring function was different for each target (Yuriev et al., 2015). The best ranking value of the six scoring functions (Chemgauss4[13], PLP[14], Chemscore[15], London dG[16], Affinity dG[16,] and Alpha HB[16]) was used as the docking score. The compounds that ranked among the top 2% were analyzed for further selection.

2.4 Selection of compounds based on the docking results

We used two methods for selecting compounds based on the docking results as follows: i) score and ii) visual inspection. The number of compounds selected based on the two methods was approximately the same.

2.4.1 By score

The compounds from step **2.3** were clustered using Bayon[17] with the GraphSim[18] fingerprint descriptor. The compound with the best score (i.e., the lowest score) from each cluster was selected.

2.4.1.1 By visual inspection

After retrieving the top ranked compounds from each cluster (step **2.3**), compounds were selected in proportion to each cluster size for the visual inspection of the remaining compounds. Individually, three members of the SBDD group inspected the 3D structures of compounds docked with the target protein and selected compounds that showed reasonable binding.

2.5 Final compound list for synthesis

For helix, strand-and-turn, and motif mimetics, approximately 8,000, 6,000, and 8,000 compounds, respectively, were selected for the subsequent synthetic process.

3 Selection of non-flat compounds

Compounds with a non-flat structure were extracted from the K-Library using the following processes.

3.1 Preprocessing of a virtual library (K-Library)

First, compounds with a MW range of 450 to 650 Da were extracted from the compounds that were not selected in the previous library construction. Next, only structures without enantiomers were selected to narrow the selection using Flipper. In addition, a single conformer was generated using Omega.

3.2 Collection of compounds with 3D shape similar to that of known PPI inhibitors and sphere-like compounds from the K-Library

Compounds registered in the PPI Inhibition Database (2P2Idb) and TIMBAL were included as known PPI inhibitors. Next, the 3D structures of the known PPI inhibitors were extracted from the complex structures in the PDB for 2P2Idb and generated from the single conformer in Omega (in the case of TIMBAL). A similarity search of the 3D structure was performed using ROCS[19] (Hawkins et al., 2007); the 3D structures of these known PPI-inhibiting compounds were employed in the query and the 3D structure of the compounds obtained by the K-Library pretreatment in step **3.1** were targeted for this purpose. Consequently, a compound with a structure similar to that of a known PPI inhibitory compound was obtained (Tanimoto combo score ≥ 1.0).

Furthermore, from the identified compounds, a known PPI inhibitor compound was used as a query and target of the identified compound; a similar structure search of the 2D structure was performed using the fingerprint obtained from GraphSim, and the known PPI inhibitor compound was searched. A compound with a similar 2D structure was searched by clustering using Bayon (the number of clusters was set to 3,000) and then removed if it was found to be in the same cluster as the known PPI inhibitor. For the identified compound, the PMI was calculated from the 3D structure and normalized principal moments ratios 1 and 2 (NPR1 and NPR2) were obtained; the compounds satisfying (NPR1 + NPR2) ≥ 1.35 were further selected. Finally, a path fingerprint was generated using GraphSim and clustering was performed with 3,000 clusters using Bayon based on the path fingerprint. The compound that showed the largest NPR1 + NPR2 value in each cluster was extracted as a representative of that cluster.

3.3 Additional collection of highly spherical compounds from K-Library

For compounds not selected in step **3.2** among those identified after the K-Library pretreatment in step **3.1.**, the PMI was calculated, and the compound pool was narrowed down by selecting those satisfying (NPR1 + NPR2) ≥ 1.35. Next, a fingerprint was generated using GraphSim; based on this fingerprint, clustering was performed with 3,000 clusters using Bayon. Compounds for which NPR1 + NPR2 showed the largest value were extracted from each cluster.

4 Selection of new scaffold compounds

We selected compounds with a new scaffold from the new scaffold library using the following steps.

4.1 Selection of new scaffold structures

We tried to synthesize novel building blocks (BB) to obtain unique compounds for PPIs. The template structures of the BBs were determined to be bicyclic spiro compounds as shown Figure S1, considering the synthetic accessibility and low planarity. For identifying hits, we searched the Generated Data Base (GDB)[20] (considered to be a unique-compounds database) using the OpenEye toolkit. The known ring systems from the ChEMBL (version 22)[21], ZINC15[22], and PubChem[23] databases were removed from the hits. The 12 candidate BB structures shown in Figure S2 were selected by visual inspection (mainly chemical stability and synthetic accessibility), followed by the removal of known substructures using the substructure search of SciFinder[24].

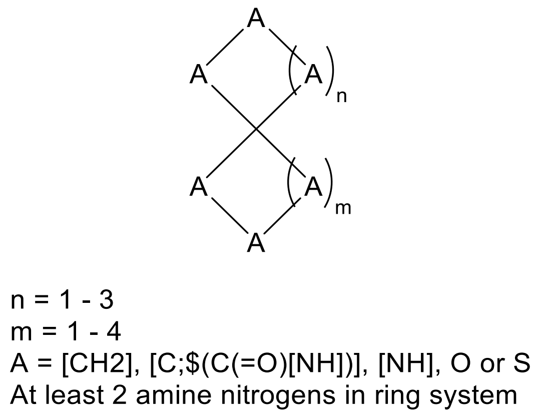

Figure S1. Bicyclic spiro structures employed for identifying novel building blocks

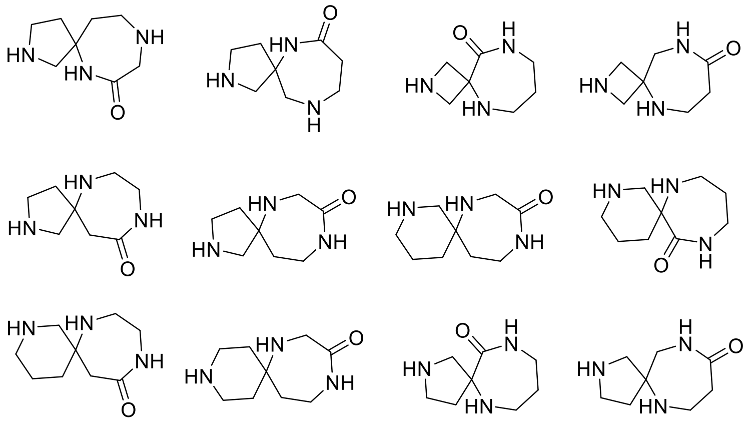

Figure S2. Building block substructures used for library synthesis

4.2 Extraction of one-point-bound compounds (two connected scaffolds)

We filtered compounds with a MW ≥ 400 from the new scaffold library. Then, a 3D structure (as a single conformer) was generated for each compound using Omega. The PMI was calculated and the top 1,500 compounds with the highest NPR1 + NPR2 were extracted.

4.3 Extraction of two-point-bound compounds (three connected scaffolds)

A substructure search of the 1,500 compounds that were identified was performed using the one-point-bound compound as a query run for compounds with three bonded scaffolds (two-point-bound

compounds) as a target. The 3D structures (single conformer) were generated using Omega for the resulting compounds, the PMI was calculated, and then the top 500 compounds with the highest NPR1 + NPR2 were extracted.

5 Synthesis of compounds

Finally, a total of 15,214 compounds were successfully synthesized and registered in the PPI compound library, as follows: helix, 4908 compounds; turn-and-strand, 2,566 compounds; and motif mimetics, 4,511 compounds; non-flat structure, 2,280; and new-scaffold structure, 949 compounds.

Table S1. List of programs, tools, and databases used in this study

No	Name	Type	Developer	URL
1	PDB	Database	RCSB PDB	https://www.rcsb.org/
2	ELM	Database	The ELM consortium	http://elm.eu.org/searchdb.html
3	Pfam	Database	EMBL-EBI	https://pfam.xfam.org/
4	MOE	Software or Program	Chemical Computing Group	http://www.chemcomp.com/
5	OEDocking TK for making grid	Software or Program	OpenEye	https://docs.eyesopen.com/toolkits/python/dockingtk/receptor.html#negative-image
6	Fsp3	Software or Program	OpenEye	https://docs.eyesopen.com/toolkits/python/molproptk/OEMolPropFunctions/OEGetFractionCsp3.html
7	PAINS filtering	Software or Program	OpenEye	https://docs.eyesopen.com/toolkits/python/molproptk/molprops.html#pains
8	Insoluble removal	Software or Program	OpenEye	https://docs.eyesopen.com/toolkits/python/molproptk/filter_files.html
9	BlockBuster filtering	Software or Program	OpenEye	https://docs.eyesopen.com/toolkits/python/molproptk/filter_files.html
10	Flipper	Software or Program	OpenEye	https://docs.eyesopen.com/toolkits/python/omegatk/OEConfGenFunctions/OEFlipper.html
11	Omega2	Software or Program	OpenEye	https://docs.eyesopen.com/toolkits/python/omegatk/index.html

12	FRED	Software or Program	OpenEye	https://docs.eyesopen.com/oedocking/fred.html
13	Chemgauss4	Software or Program	OpenEye	https://docs.eyesopen.com/toolkits/python/dockingtk/scoring.html#section-scoring-chemgauss4
14	PLP	Software or Program	OpenEye	https://docs.eyesopen.com/toolkits/python/dockingtk/scoring.html#section-scoring-plp
15	Chemscore	Software or Program	OpenEye	https://docs.eyesopen.com/toolkits/python/dockingtk/scoring.html#section-scoring-plp
16	London dG, Affinity dG, Alpha HB	Software or Program	Chemical Computing Group	https://www.chemcomp.com/MOE-Structure_Based_Design.htm
17	Bayon	Software or Program	Free Tool	https://code.google.com/archive/p/bayon/wikis/Tutorial_ja.wiki
18	OpenEye GraphSim Toolkit	Software or Program	OpenEye	https://docs.eyesopen.com/toolkits/python/graphsimtk/index.html
19	ROCS 3.3.2.2	Software or Program	OpenEye	https://www.eyesopen.com/rocs
20	GDB	Database	University of Berne	http://gdb.unibe.ch/downloads/
21	ChEMBL	Database	EMBL-EBI	https://www.ebi.ac.uk/chembl/
22	ZINC15	Database	University of California San Francisco	https://zinc15.docking.org/
23	PubChem	Database	NIH	https://pubchem.ncbi.nlm.nih.gov/
24	Scifinder	Database	CAS	https://scifinder.cas.org/

Table S2. PDB list of PPI complex structures for helix interface.

PDB ID	peptide chain	peptide name	receptor-protein chain	receptor-protein name
1dva	1dva_X	synthetic peptide	1dva_H	Coagulation factor VII
1hqq	1hqq_G	synthetic peptide	1hqq_C	Streptavidin

1nx1	1nx1_C	Calpastatin	1nx1_A	Calpain small subunit 1
1rbf	1rbf_S	Ribonuclease pancreatic	1rbf_A	Ribonuclease S
1t0j	1t0j_C	Voltage-dependent L-type calcium channel subunit alpha-1C	1t0j_B	Voltage-gated calcium channel subunit beta2a
1t3l	1t3l_B	Voltage-dependent L-type calcium channel subunit alpha-1S	1t3l_A	Voltage-dependent L-type calcium channel subunit beta-2
1y3a	1y3a_F	synthetic peptide	1y3a_B	Guanine nucleotide-binding protein G(i) subunit alpha-1
1yp0	1yp0_B	Nuclear receptor subfamily 0 group B member 2	1yp0_A	Steroidogenic factor 1
2auc	2auc_D	Myosin-A	2auc_C	Myosin A Tail Interacting Protein
2c23	2c23_P	Exoenzyme S	2c23_A	14-3-3 protein beta/alpha
2fx7	2fx7_P	Envelope glycoprotein gp160	2fx7_H	Fab 4E10
2g30	2g30_P	Low density lipoprotein receptor adapter protein 1	2g30_A	AP-2 complex subunit beta
2h1c	2h1c_B	Antitoxin FitA	2h1c_A	Toxin FitB
2nm1	2nm1_B	Synaptotagmin-2	2nm1_A	Botulinum neurotoxin type B
2pmc	2pmc_F	Protein phosphatase CheZ	2pmc_B	Chemotaxis protein CheY
2pv2	2pv2_E	synthetic peptide	2pv2_A	Chaperone SurA
2r7g	2r7g_B	Early E1A protein	2r7g_A	Retinoblastoma-associated protein
2uz6	2uz6_L	Alpha-conotoxin TXIA(A10L)	2uz6_B	Soluble acetylcholine receptor
2vzd	2vzd_C	Paxillin	2vzd_A	Alpha-parvin
2x72	2x72_B	Guanine nucleotide-binding protein G(t) subunit alpha-1	2x72_A	Rhodopsin
2xxm	2xxm_T	synthetic peptide	2xxm_A	Capsid protein P24
2xyi	2xyi_B	Histone H4	2xyi_A	Probable histone-binding protein Caf1
2y9q	2y9q_B	MAP kinase-interacting serine/threonine-protein kinase 1	2y9q_A	Mitogen-activated protein kinase 1

2z5t	2z5t_Q	Cellular tumor antigen p53	2z5t_N	Protein Mdm4
3ax3	3ax3_F	Aldehyde dehydrogenase, mitochondrial	3ax3_E	Mitochondrial import receptor subunit TOM20 homolog
3bev	3bev_C	Hemoglobin subunit alpha-A	3bev_A	MHC class I alpha chain 2
3bl2	3bl2_D	Beclin-1	3bl2_B	Bcl-2 homolog
3d32	3d32_C	synthetic peptide	3d32_A	Gamma-aminobutyric acid receptor-associated protein
3ds4	3ds4_T	synthetic peptide	3ds4_A	HIV-1 capsid protein
3gm1	3gm1_F	Paxillin	3gm1_A	Protein-tyrosine kinase 2-beta
3i1h	3i1h_B	Bcl-2 homologous antagonist/killer	3i1h_A	Bcl-2-related protein A1
3ik5	3ik5_B	T-cell surface glycoprotein CD3 zeta chain	3ik5_A	Protein Nef
3k48	3k48_R	synthetic peptide	3k48_AD	Tumor necrosis factor ligand superfamily member 13
3lnz	3lnz_D	synthetic peptide	3lnz_C	E3 ubiquitin-protein ligase Mdm2
3lrh	3lrh_F	Huntingtin	3lrh_E	Anti-huntingtin VL domain
3p72	3p72_B	synthetic peptide	3p72_A	Platelet glycoprotein Ib alpha chain
3q95	3q95_D	Nuclear receptor coactivator 2	3q95_B	Estrogen receptor
3qis	3qis_B	Sesquipedalian-1	3qis_A	Inositol polyphosphate 5-phosphatase OCRL-1
3rdv	3rdv_E	SLAIN motif-containing protein 2	3rdv_A	CAP-Gly domain-containing linker protein 1
3uvk	3uvk_B	Histone-lysine N-methyltransferase 2D	3uvk_A	WD repeat-containing protein 5
3uym	3uym_D	Type II secretion system protein D	3uym_B	Lipoprotein OutS
3zqg	3zqg_C	Anti-inducer peptide TAP2	3zqg_A	Tetracycline repressor protein class B from transposon Tn10
4ch9	4ch9_C	Serine/threonine-protein kinase WNK4	4ch9_A	Kelch-like protein 3
4fjo	4fjo_B	DNA polymerase kappa	4fjo_A	DNA repair protein REV1

4j2c	4j2c_B	Vacuolar protein sorting-associated protein 51 homolog	4j2c_A	Syntaxin-6
4j8s	4j8s_B	Tristetraprolin	4j8s_A	CCR4-NOT transcription complex subunit 1
4k0u	4k0u_B	Type II secretion system protein D	4k0u_A	Lipoprotein OutS
4nb3	4nb3_D	ATR-interacting protein	4nb3_B	Replication protein A 70 kDa DNA-binding subunit
5dhf	5dhf_D	Serine/threonine-protein kinase RIO2	5dhf_C	Exportin-1

Table S3. PDB list of PPI complex structures for turn-and-strand interface.

PDB ID	peptide chain	peptide name	receptor-protein chain	receptor-protein name
1clv	1clv_I	PROTEIN (ALPHA-AMYLASE INHIBITOR)	1clv_A	PROTEIN (ALPHA-AMYLASE)
1dkd	1dkd_F	12-MER PEPTIDE	1dkd_B	GROEL
1i8i	1i8i_C	EPIDERMAL GROWTH FACTOR RECEPTOR, EGFRVIII PEPTIDE ANTIGEN	1i8i_B	EPIDERMAL GROWTH FACTOR RECEPTOR ANTIBODY MR1SCFV HEAVY CHAIN
1jmt	1jmt_B	SPLICING FACTOR U2AF 65 KDA SUBUNIT	1jmt_A	SPLICING FACTOR U2AF 35 KDA SUBUNIT
1r9n	1r9n_H	Neuropeptide Y	1r9n_D	Dipeptidyl peptidase IV
2i60	2i60_M	[PHE23]M47, SCORPION-TOXIN MIMIC OF CD4	2i60_G	EXTERIOR MEMBRANE GLYCOPROTEIN(GP120)
2peh	2peh_D	Splicing factor 3B subunit 1	2peh_B	Splicing factor 45
2q6g	2q6g_D	Polypeptide chain	2q6g_B	severe acute respiratory syndrome coronavirus (SARS-CoV)
2w2u	2w2u_D	CONSERVED ARCHAEAL PROTEIN	2w2u_B	HYPOTHETICAL P60 KATANIN
2zvl	2zvl_U	DNA polymerase kappa	2zvl_A	Proliferating cell nuclear antigen
3lqj	3lqj_T	Histone H3	3lqj_B	MLL1 PHD3-Bromo

3m53	3m53_B	TAF10 peptide	3m53_A	Histone-lysine N-methyltransferase SETD7
3mmg	3mmg_C	Nuclear inclusion protein B fragment	3mmg_A	Nuclear inclusion protein A
3wnf	3wnf_D	CKIDNC peptide	3wnf_AB	Gag-Pol polyprotein
4bxf	4bxf_C	60S RIBOSOMAL PROTEIN L27A	4bxf_A	BIFUNCTIONAL LYSINE-SPECIFIC DEMETHYLASE AND HISTIDYL-HYDROXYLASE MINA
4dcb	4dcb_F	Plasminogen	4dcb_A	Coagulase/fibrinolysin
4gq6	4gq6_B	Histone-lysine N-methyltransferase MLL	4gq6_A	Menin
4gqb	4gqb_C	Histone H4 peptide	4gqb_A	Protein arginine N-methyltransferase 5
4h3h	4h3h_F	Pol II CTD peptide	4h3h_E	RNA polymerase II subunit A C-terminal domain phosphatase SSU72
4jzw	4jzw_M	CD4-MIMETIC MINIPROTEIN M48U1	4jzw_G	HIV-1 YU2 gp120 glycoprotein
4odm	4odm_K	30S ribosomal protein S2	4odm_D	Peptidyl-prolyl cis-trans isomerase SlyD
4oyk	4oyk_C	Ubiquitin thioesterase otulin	4oyk_A	E3 ubiquitin-protein ligase RNF31
4pr5	4pr5_C	Epstein-Barr nuclear antigen 1	4pr5_A	HLA class I histocompatibility antigen, B-35 alpha chain
4qae	4qae_T	Hepcidin	4qae_E	Neutrophil gelatinase-associated lipocalin
4yiz	4yiz_F	Rhoptry neck protein 2, putative	4yiz_E	Apical membrane antigen AMA1
4z80	4z80_D	Cytoadherence-linked asexual protein	4z80_B	EGF family domain-containing protein
5cx3	5cx3_G	FYVE and coiled-coil domain-containing protein 1	5cx3_C	Microtubule-associated proteins 1A/1B light chain 3A
5fjz	5fjz_R	PROTEIN TRANSPORT PROTEIN DSL1	5fjz_C	COATOMER SUBUNIT DELTA
5fw5	5fw5_C	NON-STRUCTURAL PROTEIN 3	5fw5_A	RAS GTPASE-ACTIVATING PROTEIN-BINDING PROTEIN 1
5jr2	5jr2_G	APYd3 peptide	5jr2_C	Ephrin type-A receptor 4

Table S4. PDB list of PPI complex structures for motif interface.

PDB ID	peptide chain	ELM motif in peptide	receptor-protein chain	receptor-protein name
1d01	1d01_I	LIG_TRAF2_1	1d01_F	TNF receptor-associated factor 2
1ee4 *	1ee4_C	TRG_NLS_MonoExtC_3	1ee4_A	Importin subunit alpha
1jd5	1jd5_B	LIG_BIR_III_3	1jd5_A	Death-associated inhibitor of apoptosis 1
1jpl	1jpl_E	TRG_LysEnd_GGAAcLL_1	1jpl_A	ADP-ribosylation factor-binding protein GGA3
1m7e	1m7e_F	LIG_PTB_Apo_2	1m7e_C	Disabled homolog 2
1nu2	1nu2_B	LIG_PTB_Apo_2	1nu2_A	Disabled homolog 1
1nw9	1nw9_B	LIG_BIR_III_2	1nw9_A	E3 ubiquitin-protein ligase XIAP
1r1s	1r1s_F	LIG_SH2_GRB2	1r1s_E	GRB2-related adaptor protein 2
1tp3	1tp3_B	LIG_PDZ_Class_1	1tp3_A	Disks large homolog 4
1w9e	1w9e_R	LIG_PDZ_Class_2	1w9e_A	Syntenin-1
1w9o	1w9o_T	LIG_PDZ_Class_2	1w9o_A	Syntenin-1
2an6	2an6_F	LIG_SIAH_1	2an6_B	E3 ubiquitin-protein ligase SIAH1A
2cci	2cci_I	LIG_CYCLIN_1	2cci_D	Cyclin-dependent kinase 2
2he2	2he2_B	LIG_PDZ_Class_	2he2_A	Disks large homolog 2
2hqh	2hqh_E	LIG_CAP-Gly_1	2hqh_A	Dynactin subunit 1
2pg1	2pg1_L	LIG_Dynein_DLC8_1	2pg1_B	Dynein light chain 1, cytoplasmic
2wmb	2wmb_I	DOC_CYCLIN_1	2wmb_B	Cyclin-A2
2xl3	2xl3_C	LIG_WD40_WDR5_VDV_1	2xl3_A	WD repeat-containing protein 5
3e87	3e87_C	MOD_PKB_1	3e87_A	RAC-beta serine/threonine-protein kinase

3esk	3esk_B	LIG_TPR	3esk_A	Stress-induced-phosphoprotein 1
3gd1	3gd1_Z	LIG_Clathr_ClatBox_1	3gd1_I	Clathrin heavy chain 1
3gjo	3gjo_F	LIG_SxIP_EBH_1	3gjo_B	Microtubule-associated protein RP/EB family member 1
3ml4	3ml4_E	LIG_PTB_Phospho_1	3ml4_A	Protein Dok-7
3pxe	3pxe_F	LIG_BRCT_BRCA1_1	3pxe_B	Breast cancer type 1 susceptibility protein
3twt	3twt_E	DOC_ANK_TNKS_1	3twt_A	Tankyrase-2
3ua7	3ua7_E	LIG_SH3_2	3ua7_A	Tyrosine-protein kinase Fyn
3ubw	3ubw_P	LIG_14-3-3_1	3ubw_A	14-3-3 protein epsilon
3ukz *	3ukz_C	TRG_NLS_Bipartite_1	3ukz_B	Importin subunit alpha-1
3utm	3utm_C	DOC_ANK_TNKS_1	3utm_A	Tankyrase-1
3v4y	3v4y_H	LIG_PP1	3v4y_G	Serine/threonine-protein phosphatase PP1-alpha catalytic subunit
3zkf	3zkf_L	LIG_Dynein_DLC8_1	3zkf_K	Dynein light chain 1, cytoplasmic
4bld	4bld_F	LIG_SUFU_	4bld_B	Maltose-binding periplasmic protein
4eje	4eje_D	LIG_PTAP_UEV_1	4eje_B	Tumor susceptibility gene 101 protein
4i7b	4i7b_D	DEG_SIAH_1	4i7b_C	E3 ubiquitin-protein ligase SIAH1
4xc2	4xc2_E	LIG_LIR_Gen_1	4xc2_A	Gamma-aminobutyric acid receptor-associated protein
4y32	4y32_C	LIG_14-3-3_3	4y32_A	14-3-3 protein sigma
5azg	5azg_D	LIG_LIR_Gen_1	5azg_B	Protein lgg-1
5d94	5d94_B	LIG_LIR_Gen_1	5d94_A	Microtubule-associated proteins 1A/1B light chain 3B

* The PDB entries have two distantly-located pockets on the peptide/protein interface of the PPI complex structure.

Supplementary Table 1. List of references published that were used to extract PPI-related compound data.

No	Reference information
1	Hwang DJ, He Y, Ponnusamy S, Mohler ML, Thiyagarajan T, McEwan IJ, Narayanan R, Miller DD. New Generation of Selective Androgen Receptor Degraders: Our Initial Design, Synthesis, and Biological Evaluation of New Compounds with Enzalutamide-Resistant Prostate Cancer Activity. J Med Chem. 2019 Jan 24;62(2):491-511. doi: 10.1021/acs.jmedchem.8b00973. Epub 2018 Dec 26. PubMed PMID: 30525603.
2	Zaiter SS, Huo Y, Tiew FY, Gestwicki JE, McAlpine SR. Designing de Novo Small Molecules That Control Heat Shock Protein 70 (Hsp70) and Heat Shock Organizing Protein (HOP) within the Chaperone Protein-Folding Machinery. J Med Chem. 2019 Jan 24;62(2):742-761. doi: 10.1021/acs.jmedchem.8b01436. Epub 2018 Dec 20. PubMed PMID: 30507174.
3	Yang X, Zhong J, Zhang Q, Qian J, Song K, Ruan C, Xu J, Ding K, Zhang J. Rational Design and Structure Validation of a Novel Peptide Inhibitor of the Adenomatous-Polyposis-Coli (APC)-Rho-Guanine-Nucleotide-Exchange-Factor-4 (Asef) Interaction. J Med Chem. 2018 Sep 13;61(17):8017-8028. doi: 10.1021/acs.jmedchem.8b01112. Epub 2018 Aug 20. PubMed PMID: 30095910.
4	He S, Dong G, Wu S, Fang K, Miao Z, Wang W, Sheng C. Small Molecules Simultaneously Inhibiting p53-Murine Double Minute 2 (MDM2) Interaction and Histone Deacetylases (HDACs): Discovery of Novel Multitargeting Antitumor Agents. J Med Chem. 2018 Aug 23;61(16):7245-7260. doi: 10.1021/acs.jmedchem.8b00664. Epub 2018 Aug 9. PubMed PMID: 30045621.
5	Cheng H, Linhares BM, Yu W, Cardenas MG, Ai Y, Jiang W, Winkler A, Cohen S, Melnick A, MacKerell A Jr, Cierpicki T, Xue F. Identification of Thiourea-Based Inhibitors of the B-Cell Lymphoma 6 BTB Domain via NMR-Based Fragment Screening and Computer-Aided Drug Design. J Med Chem. 2018 Sep 13;61(17):7573-7588. doi: 10.1021/acs.jmedchem.8b00040. Epub 2018 Jul 17. PubMed PMID: 29969259; PubMed Central PMCID: PMC6334293.
6	Roca C, Martinez-González L, Daniel-Mozo M, Sastre J, Infantes L, Mansilla A, Chaves-Sanjuan A, González-Rubio JM, Gil C, Cañada FJ, Martinez A, Sanchez-Barrena MJ, Campillo NE. Deciphering the Inhibition of the Neuronal Calcium Sensor 1 and the Guanine Exchange Factor Ric8a with a Small Phenothiazine Molecule for the Rational Generation of Therapeutic Synapse Function Regulators. J Med Chem. 2018 Jul 26;61(14):5910-5921. doi: 10.1021/acs.jmedchem.8b00088. Epub 2018 Jul 17. PubMed PMID: 29966094.
7	Shao H, Li X, Moses MA, Gilbert LA, Kalyanaraman C, Young ZT, Chernova M, Journey SN, Weissman JS, Hann B, Jacobson MP, Neckers L, Gestwicki JE. Exploration of Benzothiazole Rhodacyanines as Allosteric Inhibitors of Protein-Protein Interactions with Heat Shock Protein 70 (Hsp70). J Med Chem. 2018 Jul 26;61(14):6163-6177. doi: 10.1021/acs.jmedchem.8b00583. Epub 2018 Jul 13. PubMed PMID: 29953808; PubMed Central PMCID: PMC6104643.
8	Wang F, Jeon KO, Salovich JM, Macdonald JD, Alvarado J, Gogliotti RD, Phan J, Olejniczak ET, Sun Q, Wang S, Camper D, Yuh JP, Shaw JG, Sai J, Rossanese OW, Tansey WP, Stauffer SR, Fesik SW. Discovery of Potent 2-Aryl-6,7-dihydro-5 H-pyrrolo[1,2- a]imidazoles as WDR5-WIN-Site Inhibitors Using Fragment-Based Methods and Structure-Based Design. J Med Chem. 2018 Jul 12;61(13):5623-5642. doi: 10.1021/acs.jmedchem.8b00375. Epub 2018 Jun 29. PubMed PMID: 29889518; PubMed Central PMCID: PMC6842305.
9	Borkin D, Klossowski S, Pollock J, Miao H, Linhares BM, Kempinska K, Jin Z,Purohit T, Wen B, He M, Sun D, Cierpicki T, Grembecka J. Complexity of Blocking Bivalent Protein-Protein Interactions: Development of a Highly Potent Inhibitor of the Menin-Mixed-Lineage Leukemia Interaction. J Med Chem. 2018 Jun 14;61(11):4832-4850. doi: 10.1021/acs.jmedchem.8b00071. Epub 2018 May 23. PubMed PMID: 29738674; PubMed Central PMCID: PMC7029623.
10	Zhang M, Wang Z, Zhang Y, Guo W, Ji H. Structure-Based Optimization of Small-Molecule Inhibitors for the β-Catenin/B-Cell Lymphoma 9 Protein-Protein Interaction. J Med Chem. 2018 Apr 12;61(7):2989-3007. doi: 10.1021/acs.jmedchem.8b00068. Epub 2018 Mar 29. PubMed PMID: 29566337.
11	Hammill JT, Scott DC, Min J, Connelly MC, Holbrook G, Zhu F, Matheny A, Yang L, Singh B, Schulman BA, Guy RK. Piperidinyl Ureas Chemically Control Defective in Cullin Neddylation 1 (DCN1)-Mediated Cullin Neddylation. J Med Chem. 2018 Apr 12;61(7):2680-2693. doi: 10.1021/acs.jmedchem.7b01277. Epub 2018 Mar 26. PubMed PMID: 29547696; PubMed Central PMCID: PMC5898815.

12	Hammill JT, Bhasin D, Scott DC, Min J, Chen Y, Lu Y, Yang L, Kim HS, Connelly MC, Hammill C, Holbrook G, Jeffries C, Singh B, Schulman BA, Guy RK. Discovery of an Orally Bioavailable Inhibitor of Defective in Cullin Neddylation 1 (DCN1)-Mediated Cullin Neddylation. J Med Chem. 2018 Apr 12;61(7):2694-2706. doi: 10.1021/acs.jmedchem.7b01282. Epub 2018 Mar 26. PubMed PMID: 29547693; PubMed Central PMCID: PMC5914176.
13	Chen L, Zhuang C, Lu J, Jiang Y, Sheng C. Discovery of Novel KRAS-PDEδ Inhibitors by Fragment-Based Drug Design. J Med Chem. 2018 Mar 22;61(6):2604-2610. doi: 10.1021/acs.jmedchem.8b00057. Epub 2018 Mar 13. PubMed PMID: 29510040.
14	Zhou H, Zhou W, Zhou B, Liu L, Chern TR, Chinnaswamy K, Lu J, Bernard D, Yang CY, Li S, Wang M, Stuckey J, Sun Y, Wang S. High-Affinity Peptidomimetic Inhibitors of the DCN1-UBC12 Protein-Protein Interaction. J Med Chem. 2018 Mar 8;61(5):1934-1950. doi: 10.1021/acs.jmedchem.7b01455. Epub 2018 Feb 26. PubMed PMID: 29438612.

Supplementary Table 2. Basic statistics of physicochemical properties of protein–protein interaction (PPI) compounds by datasets in DLiP database (Small-molecule approved drugs, DLiP-PPI library, known PPI active or inactive compounds). The mean (Mean), minimum (Min), maximum (Max), and percentiles values (Percentile 25, 50 and 75) of physicochemical properties such as molecular weight (Molecular_Weight), ALogP, number of hydrogen acceptors (Num_H_Acceptors), number of hydrogen donors (Num_H_Donors), number of rotatable bonds (Num_RotatableBonds), number of rings (Num_Rings), polar surface area (PSA). The fraction of sp3 carbon atoms (FSp3), drug-likeness score (QED) were calculated using the BIOVIA Pipeline Pilot.

Small-molecule approved drugs (Molecular Weight < 1,000), 2081 compounds						
Property Name	Mean	Min	Max	Percentile25	Percentile50	Percentile75
Molecular_Weight	358.04	30.006	985.12	257.37	339.47	434.54
ALogP	2.18	-11.1	10.568	0.73	2.376	3.865
Num_H_Acceptors	5.8328	0	20	3	5	8
Num_H_Donors	2.0303	0	18	1	2	3
Num_RotatableBonds	5.3825	0	37	3	5	7
Num_Rings	2.863	0	11	2	3	4
PSA	89.049	0	368.23	46.53	78.32	115.57
Fsp3	0.4507	0	1	0.27273	0.42857	0.61414
QED	0.55367	0.04228	0.94437	0.4027	0.57116	0.72829
DLiP library (15,214 compounds)						
Property Name	Mean	Min	Max	Percentile25	Percentile50	Percentile75
Molecular_Weight	491.28	396.44	649.82	458.55	473.4	500.61
ALogP	3.0309	-2.817	8.175	2.158	3.162	4.0048
Num_H_Acceptors	6.5016	2	14	5	6	8
Num_H_Donors	1.5655	0	6	1	1	2

Num_RotatableBonds	5.8735	1	14	5	6	7
Num_Rings	4.5592	3	8	4	4	5
PSA	78.808	23.55	196.56	59.59	76.245	95.74
Fsp3	0.46284	0.05	0.77778	0.42308	0.45833	0.5
QED	0.59903	0.10352	0.85202	0.54415	0.62356	0.68488
Known PPI data, active (10,568 compounds)						
Property Name	Mean	Min	Max	Percentile25	Percentile50	Percentile75
Molecular_Weight	523.91	98.103	4049.5	409.48	494.62	580.56
ALogP	3.5834	-51.025	15.958	2.384	3.9855	5.3223
Num_H_Acceptors	8.3178	0	107	6	7	9
Num_H_Donors	2.7853	0	71	1	2	3
Num_RotatableBonds	8.5148	0	133	5	8	10
Num_Rings	3.9853	0	13	3	4	5
PSA	124.54	0	2527.8	77.92	104.06	140.42
Fsp3	0.33235	0	1	0.21053	0.32258	0.45
QED	0.40587	0.00574	0.94576	0.2559	0.3927	0.54893
Known PPI data, inactive (7,606 compounds)						
Property Name	Mean	Min	Max	Percentile25	Percentile50	Percentile75
Molecular_Weight	403.38	74.085	3422.1	295.3	373.4	468.67
ALogP	3.1896	-30.72	15.041	2.247	3.431	4.507
Num_H_Acceptors	6.5246	0	88	4	6	8

Num_H_Donors	2.2835	0	65	1	2	3
Num_RotatableBonds	6.234	0	119	3	5	8
Num_Rings	3.3396	0	13	2	3	4
PSA	102.07	0	1499.5	63.057	86.405	118.32
Fsp3	0.26639	0	1	0.10714	0.23077	0.38095
QED	0.51476	0.01095	0.94406	0.33983	0.53149	0.70414

Supplementary Figure 2. Frequent rings or scaffold structures found in protein–protein interaction (PPI) library compounds in the DLiP database. The top 20 ring structures without benzene are shown in order of frequency.

Molecule	Rank	Canonical_Smiles	Frequency
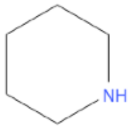	1	C1CCNCC1	12226
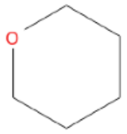	2	C1CCOCC1	5893
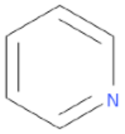	3	c1ccncc1	2391
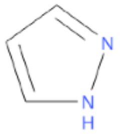	4	c1cn[nH]c1	1983
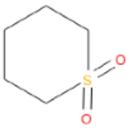	5	O=S1(=O)CCCCC1	1953
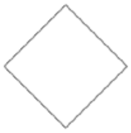	6	C1CCC1	1750
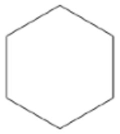	7	C1CCCCC1	1494
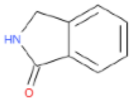	8	O=C1NCc2ccccc12	1244
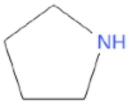	9	C1CCNC1	1075
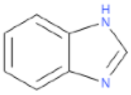	10	c1ccc2[nH]cnc2c1	983

Molecule	Rank	Canonical_Smiles	Frequency
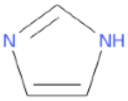	11	c1c[nH]cn1	778
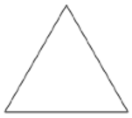	12	C1CC1	629
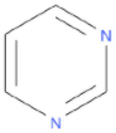	13	c1cncnc1	520
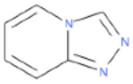	14	c1ccn2cnnc2c1	468
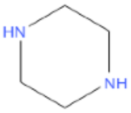	15	C1CNCCN1	461
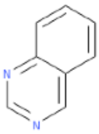	16	c1ccc2ncncc2c1	421
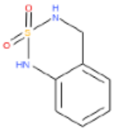	17	O=S1(=O)NCc2ccccc2N1	388
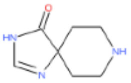	18	O=C1NC=NC12CCNCC2	319
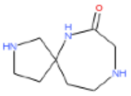	19	O=C1CNCCC2(CCNC2)N1	318
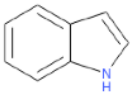	20	c1ccc2[nH]ccc2c1	266

Supplementary Figure 2. Frequent ring structures found in known protein–protein interaction (PPI) modulators in the DLiP database. The top 20 ring structures without benzene are shown in order of frequency.

Molecule	Rank	Canonical_Smiles	Frequency
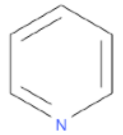	1	c1ccncc1	1417
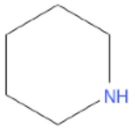	2	C1CCNCC1	1070
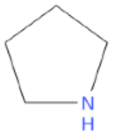	3	C1CCNC1	952
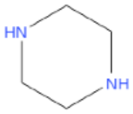	4	C1CNCCN1	745
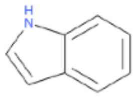	5	c1ccc2[nH]ccc2c1	603
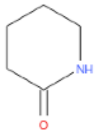	6	O=C1CCCCN1	510
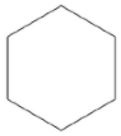	7	C1CCCCC1	500
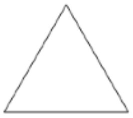	8	C1CC1	433
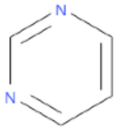	9	c1cncnc1	352
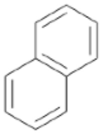	10	c1ccc2ccccc2c1	338

Molecule	Rank	Canonical_Smiles	Frequency
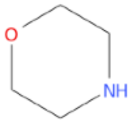	11	C1COCCN1	302
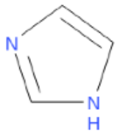	12	c1c[nH]cn1	261
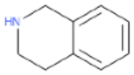	13	C1Cc2ccccc2CN1	229
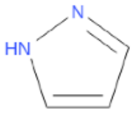	14	c1cn[nH]c1	227
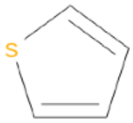	15	c1ccsc1	219
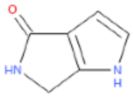	16	O=C1NCc2[nH]ccc12	216
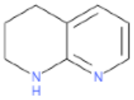	17	C1CNc2ncccc2C1	207
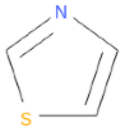	18	c1cscn1	202
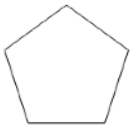	19	C1CCCC1	174
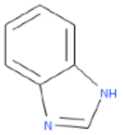	20	c1ccc2[nH]cnc2c1	164

Supplementary Figure 3. The database schema of DLiP with eight tables. CompoundProperty, CompoundStructure, MoleculeDictionary, Activity, Assay, TargetDictionary, PPIIDLookup and PPIActivity. For example, the CompoundProperty and the CompoundStructure tables are linked by the PPI compound ID.

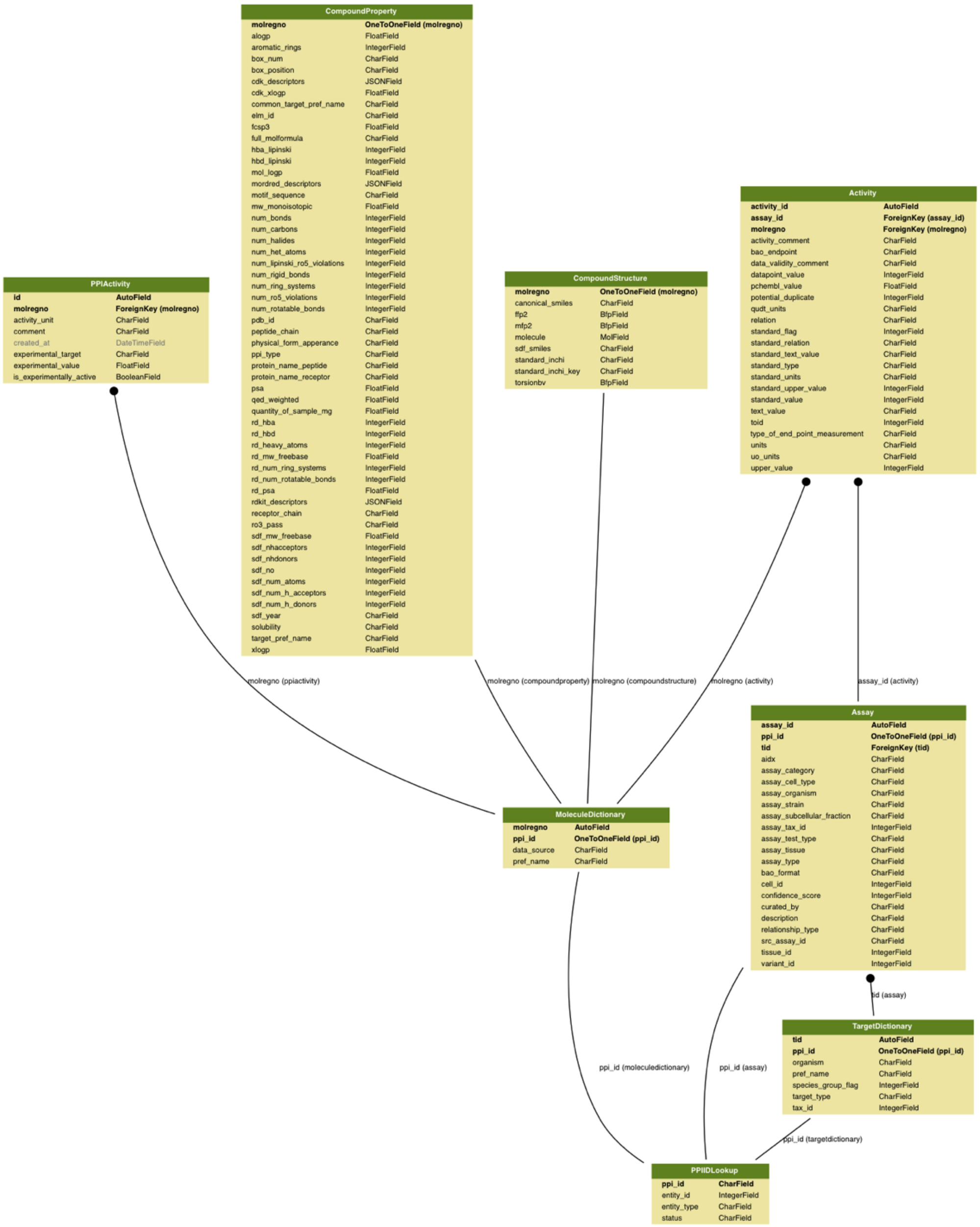

Supplement: Supplementary file 1 [file DataSheet1.PDF]
